# The biogenesis and biological functions of circular RNAs and their molecular diagnostic values in cancers

**DOI:** 10.1002/jcla.23049

**Published:** 2019-09-25

**Authors:** Haiyan Zhang, Yijing Shen, Zhe Li, Yao Ruan, Tianwen Li, Bingxiu Xiao, Weiliang Sun

**Affiliations:** ^1^ Department of Biochemistry and Molecular Biology Zhejiang Key Laboratory of Pathophysiology Medical School of Ningbo University Ningbo China; ^2^ Ningbo Yinzhou People’s Hospital and the Affiliated Hospital Medical School of Ningbo University Ningbo China

**Keywords:** biomarkers, circRNAs, diagnosis, gene expression, RT‐qPCR

## Abstract

**Background:**

In addition to non‐coding RNAs (lncRNAs) and microRNAs (miRNAs), circular RNAs (circRNAs) are endogenous RNAs with various functions, which have recently become a research hotspot. CircRNAs are a kind of closed circular RNA molecule widely existing in transcriptomes. Due to lack of free ends, they are not easily cleaved by RNase R, thus avoiding degradation. They are more stable than linear RNAs.

**Methods:**

Data were collected through PubMed. The following search terms were used: “circular RNA,” “circRNA,” “cancer,” “mechanism,” “biogenesis,” “biomarker,” “diagnosis.” Only articles published in English were included.

**Results:**

Most circRNAs express tissue/developmental stage specificity. Moreover, circRNAs are involved in the regulation of a variety of biological activities. In this review, we discuss the formation, classification, and biological functions of circRNAs, especially their molecular diagnostic values in common cancers, including gastric cancer (hsa_circ_002059, circ_LARP4, hsa_circ_0000190, hsa_circ_0000096, circ‐SFMBT2, and circ_PVT1), hepatocellular carcinoma (circ_104075, circRNA_100338, circ_MTO1, and circZKSCAN1), colorectal cancer (hsa_circ_0136666 and hsa_circ_0000523), lung cancer (hsa_circ_0006427, circ_100876, and circ_ABCB10), breast cancer (hsa_circ_0089105, circAGFG1, and circEPSTI1), bladder cancer (circFNDC3B and circTFRC), and esophageal squamous cell carcinoma (circ_100876 and circ‐DLG1).

**Conclusion:**

CircRNAs not only play important roles in tumorigenesis, but also may become new diagnostic biomarkers.

## 1 INTRODUCTION

Circular RNAs (circRNAs) are a special type of endogenous RNA molecules that are widely present in mammalian transcriptomes and involved in the regulation of gene expression. CircRNAs were first discovered in the 1970s in RNA viruses. In 1979, using an electron microscope, Hsu and Coca‐Prados first observed that some RNAs present in the cytoplasm of eukaryotic cells in a circular form.^1^ In 1991, Nigro et al^2^ first discovered that circRNAs come from spliced transcripts of candidate tumor suppressor genes. By 1993, some circRNAs were found in the transcripts of human cells.^3^ However, at that time, circRNAs were only considered as a type of RNAs formed by erroneous splicing of exon transcripts.^3^ As RNA sequencing (RNA‐seq) widespread application and the rapid growth of bioinformatics, more and more circRNAs have been found.^4^ A recent study found that changes in expression levels of circRNAs in body fluids are parallel to the somatic tissues and are believed to be associated with certain cancers.^5^ CircRNAs have also been found to be involved in the occurrence and development of many human diseases, such as nervous system disorders, cardiovascular and cerebrovascular diseases, diabetes, and cancers.^6^, ^7^, ^8^, ^9^ In this review, we introduce the formation, classification, and biological functions of circRNAs, especially their molecular diagnostic values in common cancers.

## 2 FORMATION AND CLASSIFICATION OF CIRCRNAS

**Figure 1:**
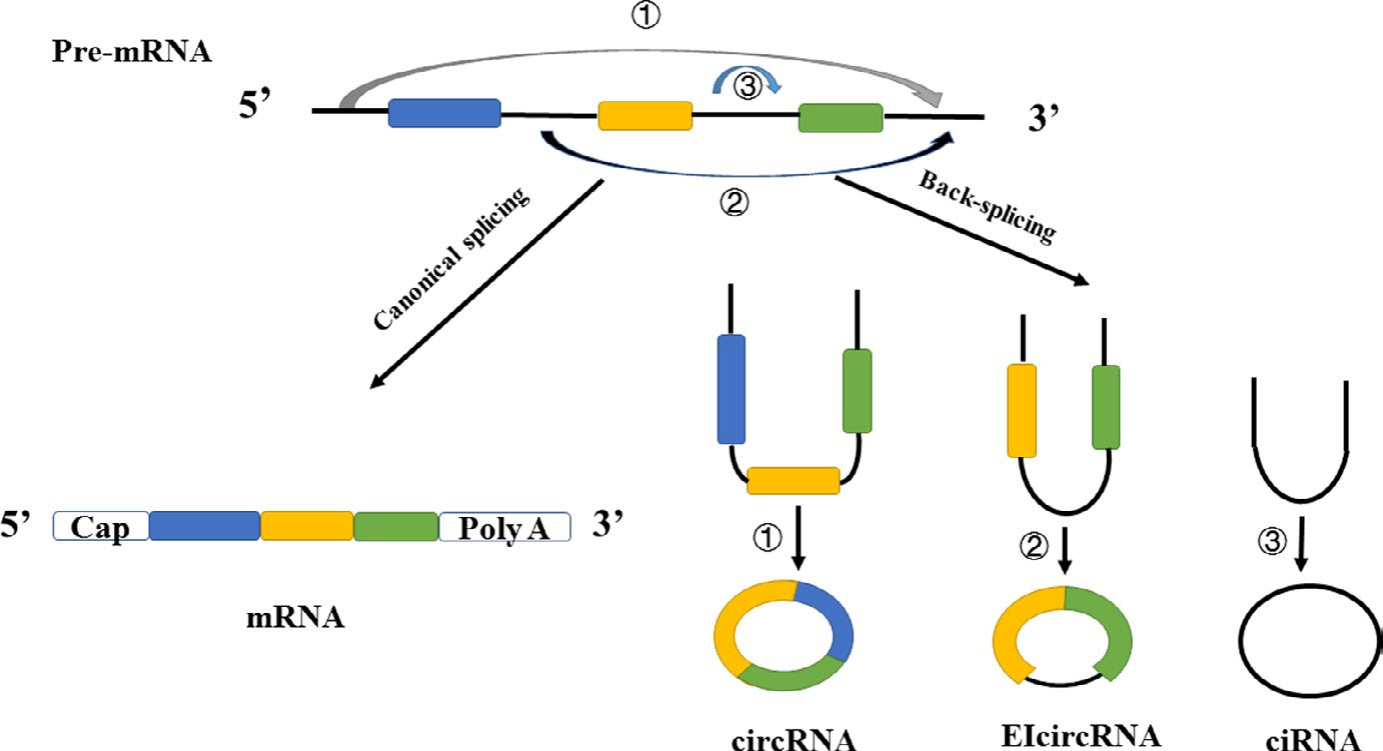
The biogenesis and classification of circular RNAs. When the pre‐mRNA is back‐spliced to produce circRNA, canonical splicing will also occur to produce mRNA

To study the possible functions of circRNAs, it is important to understand their biogenesis (Figure 1). In the past, it was thought that most human primary mRNAs are spliced into linear RNA that retain only exons. In recent years, even though the mechanisms underlying circRNA formation remain unclear, two kinds of exonic circRNA formation models, lariat‐driven circularization and intron‐pairing‐driven circularization, were proposed in 2013.^10^ Subsequently, RNA‐binding quaking (QKI), a member of the STAR family in the KH domain‐containing RNA‐binding proteins, was found to affect pre‐mRNA splicing and promote circRNA biosynthesis during epithelial‐mesenchymal transition (EMT).^11^ Additionally, the formation of circRNAs can be influenced by adenosine deaminase (ADA), an RNA‐editing enzyme that acts on RNA.^12^ It is generally believed that the reverse splicing occurs when the downstream 5′ splicing site is connected to the upstream 3′ splicing site to generate circRNAs.^13^

CircRNAs are produced by exons or introns through reverse splicing or lariat introns. However, in recent years, researchers have found that exon transcripts in pre‐mRNA can also be reverse spliced non‐linearly to form circRNAs,^10^, ^14^, ^15^ including one exon loop, two exon loops, and three or more exon loops, as well as exon‐intron hybrid loops (EIcircRNA).^16^, ^17^, ^18^, ^19^ In addition, the intron itself can also circularize and then form circRNA (ciRNA).^20^, ^21^ Among them, the exonic type is the most common type.

## 3 BIOLOGICAL FUNCTIONS OF CIRCRNAS

Although most of the biological functions of circRNA remain unclear, circRNAs have been reported that to play important roles in normal conditions and disease situations. The biological functions of circRNAs may be divided into four aspects: acting as a microRNA (miRNA) sponge, interacting with RNA‐binding proteins (RBPs), encoding proteins, and regulating transcription processes.

First, circRNAs may act as miRNA sponges. This is the most studied function of circRNAs. It is well known that the cerebellar degeneration‐associated protein 1 antisense transcript (CDR1as) contains more than 70 miR‐7‐binding sites but is not degraded by RNA‐induced silencing complex (RISC).^14^, ^22^ CDR1as is a circular inhibitor of miR‐7.^23^ When CDR1as is highly expressed, miR‐7 activity is decreased, leading to increased expression of miR‐7's target genes.^10^, ^14^, ^22^, ^24^ Therefore, CDR1as is also known as CiRS‐7, a sponge of miR‐7. We all know that disordered miRNAs may be used as oncogenes (oncomiRs) or tumor suppressor genes (ts‐miRs), which play an important role in the development of tumors.^25^, ^26^, ^27^, ^28^ miR‐21 is one of the most characteristic oncomiRs overexpressed in gastric cancer.^29^, ^30^ It has been reported that in vitro synthesized circRNA that binds to miR‐21 (scRNA21) can significantly inhibit the expression of tumor suppressor gene DAXX (a gene encoding a death domain‐associated protein), which is originally inhibited by miR‐21, thereby significantly inhibiting gastric cancer cells with high expression of miR‐21.^31^ Based on this, scRNA21 can be applied to treatment of patients with gastric cancer.

Second, circRNAs may interact with RBPs and then participate in the regulation of gene expression. RBPs are known to play a crucial role in a variety of cellular processes, such as cell function, trafficking, and localization, particularly in the post‐transcriptional regulation of RNA. Circ‐Foxo3 (generated by the Foxo‐3 gene) inhibits cell cycle progression by binding to cyclin‐dependent kinase 2 (CDK2) and cyclin‐dependent kinase inhibitor 1 (P21) to form a ternary complex, circ‐Foxo3‐p21‐CDK2.^32^ In general, CDK2 binds to cyclins A and E to promote cell cycle entry, while P21 inhibits these interactions and prevents cell cycle progression. This complex impedes the function of CDK2 and thus blocks the progression of the cell cycle.^33^

Third, some circRNAs may even encode proteins. Generally speaking, circRNAs have no ability to encode proteins, so they were once thought of as non‐coding RNAs. However, researchers have found that some circRNAs can be translated if they contain internal ribosome entry site elements (IRES)^34^ or open reading frame (ORF).^35^ These discoveries have opened new doors for circRNA studies. A report shows that SHPRH‐146aa is a new protein produced by coding of the SNF2 histone linker PHD RING helicase (*SHPRH*) gene.^36^ Circ‐SHPRH uses overlapping genetic code to produce the 'UGA' termination codon, which leads to the translation of 17 kDa SHPRH‐146aa. Circ‐SHPRH is highly expressed in normal human brain cells but is reduced in glioblastoma. High expression of SHPRH‐146aa in glioblastoma cells may reduce malignancy and tumorigenicity in vitro and in vivo.^36^ As a result, this protein can become a tumor suppressor of human glioblastoma.

Fourth, circRNAs may regulate transcription processes. Zhang et al^37^ found that circRNAs regulated the expression of their parental genes. Generally speaking, these circRNAs that regulate the transcription process are rich in the nucleus.^37^ Additionally, EIciRNAs, such as EIciEIF3J and EIciPAIP2, can bind to nuclear ribonucleoprotein with U1 small nuclear RNA (U1snRNP) and RNA polymerase II (Pol II) in a cis‐acting form to enhance transcription of their parental genes.^38^ If the interaction between RNA and RNA is blocked, the binding of EIciRNA to Pol II is reduced, and resulting in fewer EIciRNA‐U1 snRNP complexes that bind to the gene‐encoding promoter.^34^

## 4 THE POTENTIAL DIAGNOSTIC ROLES OF CIRCRNAS IN CANCERS

With regard to cancers, early detection, early diagnosis, and early treatment are the most effective ways to reduce death caused by cancers. However, many cancer patients have reached the late stage when they are clinically discovered due to the lack of early diagnostic biomarkers. CircRNAs play an important regulatory role in the occurrence and progression of diseases, especially in cancers. They are expected to become novel tumor biomarkers of diagnosis and new targets for treatment of gastric cancer,^5^, ^39^, ^40^, ^41^, ^42^ hepatocellular carcinoma,^43^, ^44^, ^45^, ^46^ colorectal cancer,^47^, ^48^ lung cancer,^49^, ^50^, ^51^ breast cancer,^52^, ^53^, ^54^ bladder cancer,^55^, ^56^ and esophageal squamous cell carcinoma.^57^, ^58^

Gastric cancer is a malignant tumor originating from the gastric mucosal epithelium and is one of the most common malignant tumors in the world. At present, the mortality rate of gastric cancer is still on the rise. When diagnosed, most patients with gastric cancer have reached the middle and late stages. Early diagnosis and early treatment are the most effective ways to reduce tumor mortality. As reported, hsa_circ_002059, hsa_circ_0000190, and circ_LARP4 expressions in gastric cancer tissues were significantly downregulated compared with those in adjacent non‐tumor tissues.^5^, ^39^, ^40^ The expression levels of hsa_circ_002059 in plasma of patients with gastric cancer were also significantly different from those before surgery.^5^ These results suggest that hsa_circ_002059 may be a novel, stable biomarker for the diagnosis of gastric cancer.^5^ CircLARP4 (La ribonucleoprotein domain family member 4) is primarily localized in the cytoplasm and inhibits proliferation of gastric cancer cells by sponge on miR‐424 and represents an independent prognostic factor for overall survival in gastric cancer patients.^39^ The area under the receiver operating characteristic (ROC) curve (AUC) of hsa_circ_0000190 in tissues and plasma was 0.75 and 0.60, respectively; and the combined AUC increased to 0.775.^40^ The sensitivity and specificity of hsa_circ_0000190 were 0.712 and 0.750, respectively. They are superior to the commonly used biomarker carcinoembryonic antigen (CEA). Therefore, they may be non‐invasive diagnostic biomarkers for gastric cancer.^40^ Other circRNAs, circ‐SFMBT2 and circ_PVT1, were found to increase expression in gastric cancer tissues.^41^, ^42^ Circ‐SFMBT2 was associated with the tumor stage of gastric cancer, and silencing circ‐SFMBT2 significantly inhibited the proliferation of gastric cancer cells.^41^ More importantly, circ‐SFMBT2 acts as a sponge for miR‐182‐5p to regulate mRNA expression of cAMP response element‐binding protein 1 (CREB1).^41^ The fact that circ‐SFMBT2 regulates the involvement of CREB1 mRNA in gastric cancer progression by competing miR‐182‐5p offers a new target for the treatment of gastric cancer. Circ_PVT1 is derived from plasmacytoma variant translocation 1 gene (PVT1), and the high expression of circPVT1 in gastric cancer tissues is due to the enlargement of its genomic locus and can promote cell proliferation through the sponge action of miR‐125.^42^ The expression level of circPVT1 can be used as an independent prognostic biomarker for gastric cancer patients in terms of overall survival (OS) and disease‐free survival.^42^ Therefore, circPVT1 may become a prognostic biomarker of gastric cancer.

Hepatocellular carcinoma (HCC) is currently the most common primary liver cancer in China. Due to the lack of early diagnostic biomarkers with high specificity and sensitivity, most HCC patients have reached an advanced disease stage when diagnosed.^59^ In recent years, experiments have found that circ_104075 and circRNA_100338 were highly expressed in HCC tissues, plasma, and cell lines.^43^, ^44^ Furthermore, the AUC of circ_104075 was 0.973 with a sensitivity of 0.96 and specificity of 0.983.^43^ These mean that circ_104075 has the potential to become a new biomarker for the diagnosis of HCC. The sponge effect of circRNA_100338 with miR‐141‐3p plays a key antagonistic role in the regulation of HCC cell invasion.^44^ The differential expression in hepatitis B‐related HCC patients shows clinical significance that circRNA_100338 may be a potentially valuable biomarker for HCC diagnosis and a target for HCC treatment.^44^ CircMTO1 (derived from mitochondrial translation optimization 1 homologue) is also called hsa_circRNA_0007874. The low expression levels of circMTO1 are related to the short survival cycle of HCC.^45^ CircMTO1 may be used as a prognostic factor for the low survival rate of patients. In addition, circMTO1 can inhibit the progression of HCC by promoting the expression of P21 by acting as a sponge for miR‐9, suggesting that circMTO1 can be a potential target for HCC treatment.^45^ CircZKSCAN1, derived from the zinc finger family gene ZKSCAN1, was found to be crucially downregulated in HCC tissues compared with non‐tumorous tissues.^46^ Further study showed that decreasing the expression of circZKSCAN1 promoted the proliferation, invasion, and distant metastasis of HCC cells.^46^ This study indicates that circZKSCAN1 may serve as a potential diagnostic biomarker for HCC.

Colorectal cancer (CRC) is one of the most common gastrointestinal tumors and one of the leading causes of cancer deaths worldwide.^60^ At present, increasing evidence shows that circRNAs impact the tumor progression of CRC. Research has shown that hsa_circ_0136666 is highly expressed in CRC tissues and cell lines, and the degree of high expression is closely related to the OS rate of CRC patients.^47^ Another circRNA, hsa_circ_0000523, was expressed at low levels in CRC tissues and cell lines.^48^ In addition, hsa_circ_0000523 acts as a "sponge" of miR‐31 and indirectly regulates the Wnt/β‐catenin signaling pathway, thereby participating in the progression of CRC.^48^

Lung cancer is one of the fastest growing malignant tumors with the highest morbidity and mortality. In the past 50 years, the incidence and mortality of lung cancer have increased, especially in men. Lung adenocarcinoma (LUAD) is considered to be the most common type of lung cancer.^61^ Despite advances in the treatment of LUAD, a complete cure remains difficult to attain.^62^ Thus, it is necessary to understand the specific pathogenesis of LUAD.^63^ Previous study has demonstrated that hsa_circ_0006427 was expressed at low levels in LUAD tissues and cell lines and was associated with prognosis,^49^ while both circ_100876 and circ_ABCB10 are highly expressed in non‐small‐cell lung cancer (NSCLC) tissues.^50^, ^51^ CircRNA_100876 is closely connected to the carcinogenesis of NSCLC.^50^ This means that circ_100876 may become a potential prognostic biomarker and therapeutic target for NSCLC.^50^ CircABCB10, also known as circRNA_0008717, promotes proliferation and distant metastasis of NSCLC cells via the miR‐1252/FOXR2 axis.^51^ This result provides a new diagnostic and therapeutic target for NSCLC.

Breast cancer (BC) is one of the leading causes of cancer‐related death in women and the most serious threat to women's health.^64^ Due to the lack of effective early diagnostic markers, the prognosis of BC treatment is very poor.^65^ Research has demonstrated that circASS1, also known as hsa_circ_0089105, is reduced in BC cell lines, and less expression of hsa_circ_0089105 promotes incursion and metastasis of BC cells.^52^ CircAGFG1 and circEPSTI1 are highly expressed in triple‐negative BC (NTBC).^53^, ^54^ The expression levels of circAGFG1 are closely related to clinical pathological stage and poor prognosis.^53^ This means that circAGFG1 may be expected to act as a new diagnostic biomarker and therapeutic target for NTBC.^53^ CircEPSTI1 (hsa_ circRNA_000479) promotes the proliferation of TNBC cells and is associated with survival in TNBC patients.^54^ It may serve as an independent prognostic biomarker for TNBC.

Bladder cancer is one of the most common malignancies of the urinary system worldwide.^66^ The expression of circFNDC3B has been found to be reduced in bladder cancer tissues and is associated with clinical pathological stage, lymph node metastasis, and OS of patients.^55^ Meanwhile, circTFRC is upregulated in bladder cancer.^56^ CircTFRC can promote the proliferation of bladder cancer cell line and tumor growth and is related to the low tumor stage and survival rate.^56^ As a result, circTFRC may serve as a new biomarker of bladder cancer.

Esophageal cancer (EC) is a common digestive tract tumor. The morbidity and mortality vary widely in different regions.^67^ A study has shown that circ_100876 is highly expressed in esophageal squamous cell carcinoma (ESCC).^57^ It can promote cell proliferation, incursion, and distal metastasis, as well as the progress of EMT.^57^ Circ‐DLG1 was observed to be increased in ESCC tissues, cell lines, and plasma and can significantly promote cell proliferation.^58^ These results illustrate that circ‐DLG1 may become a novel diagnostic biomarker of ECSS.

## 5 CONCLUSIONS AND PERSPECTIVES

Over the years, with the rapid development of widely used RNA sequencing and bioinformatics, circRNAs have drawn an increasing attention. Their structure and functions are also increasingly known. Although much progress has been made in the research on circRNAs, more in‐depth mechanism studies are needed.

Different types of circRNAs are located in different sites of cells. Exonic circRNAs are located in the cytoplasm, while some ciRNAs and EIciRNAs are located in the nucleus,^10^, ^14^, ^37^, ^68^ suggesting that circRNAs may have a variety of roles in cells. The latest study has shown that circRNAs are abundant and stable in the extracellular vesicles (EVs) and can be delivered to the exosomes.^69^ In addition, cancer cells can transport circRNAs via EVs for intercellular communication.^70^ Additionally, increasing evidence shows that circRNAs may become potential therapeutic targets for cancer patients.^71^, ^72^

It is known that for gastric cancer, CEA is the most commonly used screening biomarker.^73^ However, its sensitivity and specificity are only approximately 70% and 50%, respectively. If early gastric cancer can be found and treated immediately, the 5‐year survival rate can reach more than 90%. There have been reports about the combined use of circRNAs in the diagnosis of gastric cancer.^74^, ^75^ For example, the AUC of hsa_circ_0000096 for the diagnosis of gastric cancer is 0.82, but when combined with hsa_circ_002059, the AUC can reach 0.91.^74^

For the use of circRNAs in the treatment of cancers, recent studies have found that the in vitro synthesized miR‐21‐targeted circular RNA sponge scRNA21 can significantly inhibit the proliferation of gastric cancer cells.^31^ Another study on ESCC found that overexpression of CiRS‐7 in vitro and in vivo counteracts the ability of miR‐7 to inhibit cancer cell proliferation, incursion, and lung distal metastasis.^76^

In summary, circRNAs not only play important roles in tumor diagnosis, but also may become new targets in treating cancers (Table 1).

**Table 1:** Summary of the clinical significances of some representative circRNAs in common cancers

Cancer type	Level	circRNA	Clinical significances	Reference
Gastric cancer	Down	hsa_circ_002059	Diagnostic biomarker	5
		circ_LARP4	Suppressive effect and diagnosis	39
		hsa_circ_0000190	Non‐invasive diagnostic biomarker	40
		hsa_circ_0000096	Diagnostic biomarker	74
	Up	circ_SFNBT2	Treatment target	41
		circ_PVT1	Proliferative effect and prognostic biomarker	42
Hepatocellular carcinoma	Up	circRNA_104075	Diagnostic and treatment biomarker	43
		circRNA_100338	Diagnostic biomarker and therapeutic target	44
	Down	circ_MTO1	Therapeutic target and prognostic predictor	45
		circ_ZKSCAN1	Diagnostic biomarker	46
Colorectal cancer	Up	hsa_circ_0136666	Treatment target	47
	Down	hsa_circ_0000523	‐	48
Lung cancer	Down	hsa_circ_0006427	‐	49
	Up	circRNA_100876	Prognostic biomarker and therapeutic target	50
		circ_ABCB10	Diagnostic biomarker and therapeutic target	51
Breast cancer	Down	circASS1	‐	52
	Up	circAGFG1	Diagnostic biomarker and therapeutic target	53
		circEPSTI1	Independent prognostic biomarker	54
Bladder cancer	Down	circFNDC3B	‐	55
	Up	circTFRC	Diagnostic biomarker	56
Esophageal cancer	Up	circRNA_100876	‐	57
	Down	circ_DLG1	Diagnostic biomarker	58

## CONFLICT OF INTEREST

None.

## References

[1] Hsu MT , Coca‐Prados M . Electron microscopic evidence for the circular form of RNA in the cytoplasm of eukaryotic cells. Nature. 1979;280(5720):339‐340. 460409 10.1038/280339a0

[2] Nigro JM , Cho KR , Fearon ER , et al. Scrambled exons. Cell. 1991;64(3):607‐613. 1991322 10.1016/0092-8674(91)90244-s

[3] Cocquerelle C , Mascrez B , Hetuin D , Bailleul B . Mis‐splicing yields circular RNA molecules. FASEB J. 1993;7(1):155‐160. 7678559 10.1096/fasebj.7.1.7678559

[4] Zhao Q , Chen S , Li T , Xiao B , Zhang X . Clinical values of circular RNA 0000181 in the screening of gastric cancer. J Clin Lab Anal. 2018;32(4):e22333. 28940688 10.1002/jcla.22333 PMC6817246

[5] Li P , Chen S , Chen H , et al. Using circular RNA as a novel type of biomarker in the screening of gastric cancer. Clin Chim Acta. 2015;444:132‐136. 25689795 10.1016/j.cca.2015.02.018

[6] Rybak‐Wolf A , Stottmeister C , Glažar P , et al. Circular RNAs in the mammalian brain are highly abundant, conserved, and dynamically expressed. Mol Cell. 2015;58(5):870‐885. 25921068 10.1016/j.molcel.2015.03.027

[7] Burd CE , Jeck WR , Liu Y , Sanoff HK , Wang Z , Sharpless NE . Expression of linear and novel circular forms of an INK4/ARF‐associated non‐coding RNA correlates with atherosclerosis risk. PLoS Genet. 2010;6(12):e1001233. 21151960 10.1371/journal.pgen.1001233 PMC2996334

[8] Tian M , Chen R , Li T , Xiao B . Reduced expression of circRNA hsa_circ_0003159 in gastric cancer and its clinical significance. J Clin Lab Anal. 2018;32(3):e22281. 10.1002/jcla.22281 PMC6817154 28618205

[9] Li T , Shao Y , Fu L , et al. Plasma circular RNA profiling of patients with gastric cancer and their droplet digital RT‐PCR detection. J Mol Med (Berl). 2018;96(1):85‐96. 29098316 10.1007/s00109-017-1600-y

[10] Jeck WR , Sorrentino JA , Wang K , et al. Circular RNAs are abundant, conserved, and associated with ALU repeats. RNA. 2013;19(2):141‐157. 23249747 10.1261/rna.035667.112 PMC3543092

[11] Conn S , Pillman K , Toubia J , et al. The RNA binding protein quaking regulates formation of circRNAs. Cell. 2015;160(6):1125‐1134. 25768908 10.1016/j.cell.2015.02.014

[12] Ivanov A , Memczak S , Wyler E , et al. Analysis of intron sequences reveals hallmarks of circular RNA biogenesis in animals. Cell Rep. 2015;10(2):170‐177. 25558066 10.1016/j.celrep.2014.12.019

[13] Zhang XO , Dong R , Zhang Y , et al. Diverse alternative back‐splicing and alternative splicing landscape of circular RNAs. Genome Res. 2016;26(9):1277‐1287. 27365365 10.1101/gr.202895.115 PMC5052039

[14] Memczak S , Jens M , Elefsinioti A , et al. Circular RNAs are a large class of animal RNAs with regulatory potency. Nature. 2013;495(7441):333‐338. 23446348 10.1038/nature11928

[15] Fu L , Jiang Z , Li T , Hu Y , Guo J . Circular RNAs in hepatocellular carcinoma: Functions and implications. Cancer Med. 2018;7(7):3101‐3109. 10.1002/cam4.1574 PMC6051148 29856133

[16] Salzman J , Chen RE , Olsen MN , Wang PL , Brown PO . Cell‐type specific features of circular RNA expression. PLoS Genet. 2013;9(9):e1003777. 24039610 10.1371/journal.pgen.1003777 PMC3764148

[17] Dong Y , He D , Peng Z , et al. Circular RNAs in cancer: an emerging key player. J Hematol Oncol. 2017;10(1):2. 28049499 10.1186/s13045-016-0370-2 PMC5210264

[18] Zlotorynski E . Non‐coding RNA: circular RNAs promote transcription. Nat Rev Mol Cell Biol. 2015;16(4):206. 10.1038/nrm3967 25714680

[19] Yao T , Chen Q , Fu L , Guo J . Circular RNAs: biogenesis, properties, roles, and their relationships with liver diseases. Hepatol Res. 2017;47(6):497‐504. 28185365 10.1111/hepr.12871

[20] Rearick D , Prakash A , McSweeny A , Shepard SS , Fedorova L , Fedorov A . Critical association of ncRNA with introns. Nucleic Acids Res. 2011;39(6):2357‐2366. 21071396 10.1093/nar/gkq1080 PMC3064772

[21] Yin LF , Hu MJ , Wang F , et al. Frequent gain and loss of introns in fungal cytochrome b genes. PLoS ONE. 2012;7(11):e49096. 23145081 10.1371/journal.pone.0049096 PMC3492308

[22] Hansen TB , Jensen TI , Clausen BH , et al. Natural RNA circles function as efficient microRNA sponges. Nature. 2013;495(7441):384‐388. 23446346 10.1038/nature11993

[23] Hansen TB , Kjems J , Damgaard CK . Circular RNA and miR‐7 in cancer. Cancer Res. 2013;73(18):5609‐5612. 24014594 10.1158/0008-5472.CAN-13-1568

[24] Hentze MW , Preiss T . Circular RNAs: splicing's enigma variations. EMBO J. 2013;32(7):923‐925. 23463100 10.1038/emboj.2013.53 PMC3616293

[25] Xue L , Xie L , Song X , Song X . Identification of potential tumor‐educated platelets RNA biomarkers in non‐small‐cell lung cancer by integrated bioinformatical analysis. J Clin Lab Anal. 2018;32(7):e22450. 29665143 10.1002/jcla.22450 PMC6817076

[26] Xia T , Liao Q , Jiang X , et al. Long noncoding RNA associated‐competing endogenous RNAs in gastric cancer. Sci Rep. 2014;4:6088. 25124853 10.1038/srep06088 PMC4133709

[27] Wang C , Dong H , Fan H , Wu J , Wang G . Genetic polymorphisms of microRNA machinery genes predict overall survival of esophageal squamous carcinoma. J Clin Lab Anal. 2018;32(1):e22170. 10.1002/jcla.22170 PMC6816893 29226993

[28] Li PF , Chen SC , Xia T , et al. Non‐coding RNAs and gastric cancer. World J Gastroenterol. 2014;20(18):5411‐5419. 24833871 10.3748/wjg.v20.i18.5411 PMC4017056

[29] Cui L , Zhang X , Ye G , et al. Gastric juice MicroRNAs as potential biomarkers for the screening of gastric cancer. Cancer. 2013;119(9):1618‐1626. 23335180 10.1002/cncr.27903

[30] Guo J , Miao Y , Xiao B , et al. Differential expression of microRNA species in human gastric cancer versus non‐tumorous tissues. J Gastroenterol Hepatol. 2009;24(4):652‐657. 19175831 10.1111/j.1440-1746.2008.05666.x

[31] Liu XI , Abraham JM , Cheng Y , et al. Synthetic circular RNA functions as a miR‐21 sponge to suppress gastric carcinoma cell proliferation. Mol Ther Nucleic Acids. 2018;13:312‐321. 30326427 10.1016/j.omtn.2018.09.010 PMC6197335

[32] Anderson MJ , Viars CS , Czekay S , Cavenee WK , Arden KC . Cloning and characterization of three human forkhead genes that comprise an FKHR‐like gene subfamily. Genomics. 1998;47(2):187‐199. 9479491 10.1006/geno.1997.5122

[33] Du WW , Yang W , Liu E , Yang Z , Dhaliwal P , Yang BB . Foxo3 circular RNA retards cell cycle progression via forming ternary complexes with p21 and CDK2. Nucleic Acids Res. 2016;44(6):2846‐2858. 26861625 10.1093/nar/gkw027 PMC4824104

[34] Chen CY , Sarnow P . Initiation of protein synthesis by the eukaryotic translational apparatus on circular RNAs. Science. 1995;268(5209):415‐417. 7536344 10.1126/science.7536344

[35] Perriman R , Ares M Jr . Circular mRNA can direct translation of extremely long repeating‐sequence proteins in vivo. RNA. 1998;4(9):1047‐1054. 9740124 10.1017/s135583829898061x PMC1369681

[36] Zhang M , Huang N , Yang X , et al. A novel protein encoded by the circular form of the SHPRH gene suppresses glioma tumorigenesis. Oncogene. 2018;37(13):1805‐1814. 29343848 10.1038/s41388-017-0019-9

[37] Zhang Y , Zhang X‐O , Chen T , et al. Circular intronic long noncoding RNAs. Mol Cell. 2013;51(6):792‐806. 24035497 10.1016/j.molcel.2013.08.017

[38] Li Z , Huang C , Bao C , et al. Exon‐intron circular RNAs regulate transcription in the nucleus. Nat Struct Mol Biol. 2015;22(3):256‐264. 25664725 10.1038/nsmb.2959

[39] Zhang J , Liu H , Hou L , et al. Circular RNA_LARP4 inhibits cell proliferation and invasion of gastric cancer by sponging miR‐424‐5p and regulating LATS1 expression. Mol Cancer. 2017;16(1):151. 28893265 10.1186/s12943-017-0719-3 PMC5594516

[40] Chen S , Li T , Zhao Q , Xiao B , Guo J . Using circular RNA hsa_circ_0000190 as a new biomarker in the diagnosis of gastric cancer. Clin Chim Acta. 2017;466:167‐171. 28130019 10.1016/j.cca.2017.01.025

[41] Sun H , Xi P , Sun Z , et al. Circ‐SFMBT2 promotes the proliferation of gastric cancer cells through sponging miR‐182‐5p to enhance CREB1 expression. Cancer Manag Res. 2018;10:5725‐5734. 30510446 10.2147/CMAR.S172592 PMC6248399

[42] Chen J , Li Y , Zheng Q , et al. Circular RNA profile identifies circPVT1 as a proliferative factor and prognostic marker in gastric cancer. Cancer Lett. 2017;388:208‐219. 27986464 10.1016/j.canlet.2016.12.006

[43] Zhang X , Xu Y , Qian Z , et al. circRNA_104075 stimulates YAP‐dependent tumorigenesis through the regulation of HNF4a and may serve as a diagnostic marker in hepatocellular carcinoma. Cell Death Dis. 2018;9(11):1091. 30361504 10.1038/s41419-018-1132-6 PMC6202383

[44] Huang X‐Y , Huang Z‐L , Xu Y‐H , et al. Comprehensive circular RNA profiling reveals the regulatory role of the circRNA‐100338/miR‐141‐3p pathway in hepatitis B‐related hepatocellular carcinoma. Sci Rep. 2017;7(1):5428. 28710406 10.1038/s41598-017-05432-8 PMC5511135

[45] Han D , Li J , Wang H , et al. Circular RNA circMTO1 acts as the sponge of microRNA‐9 to suppress hepatocellular carcinoma progression. Hepatology. 2017;66(4):1151‐1164. 28520103 10.1002/hep.29270

[46] Yao Z , Luo J , Hu K , et al. ZKSCAN1 gene and its related circular RNA (circZKSCAN1) both inhibit hepatocellular carcinoma cell growth, migration, and invasion but through different signaling pathways. Mol Oncol. 2017;11(4):422‐437. 28211215 10.1002/1878-0261.12045 PMC5527481

[47] Jin C , Wang A , Liu L , Wang G , Li G . Hsa_circ_0136666 promotes the proliferation and invasion of colorectal cancer through miR‐136/SH2B1 axis. J Cell Physiol. 2019;234(5):7247‐7256. 30370521 10.1002/jcp.27482

[48] Jin Y , Yu LL , Zhang B , Liu CF , Chen Y . Circular RNA hsa_circ_0000523 regulates the proliferation and apoptosis of colorectal cancer cells as miRNA sponge. Braz J Med Biol Res. 2018;51(12):e7811. 30403259 10.1590/1414-431X20187811 PMC6233523

[49] Yao Y , Hua Q , Zhou Y . CircRNA has_circ_0006427 suppresses the progression of lung adenocarcinoma by regulating miR‐6783‐3p/DKK1 axis and inactivating Wnt/beta‐catenin signaling pathway. Biochem Biophys Res Commun. 2019;508(1):37‐45. 30470570 10.1016/j.bbrc.2018.11.079

[50] Yao JT , Zhao SH , Liu QP , et al. Over‐expression of CircRNA_100876 in non‐small cell lung cancer and its prognostic value. Pathol Res Pract. 2017;213(5):453‐456. 28343871 10.1016/j.prp.2017.02.011

[51] Tian X , Zhang L , Jiao Y , Chen J , Shan Y , Yang W . CircABCB10 promotes nonsmall cell lung cancer cell proliferation and migration by regulating the miR‐1252/FOXR2 axis. J Cell Biochem. 2019;120(3):3765‐3772. 30417418 10.1002/jcb.27657 PMC6587869

[52] Hou J‐C , Xu Z , Zhong S‐L , et al. Circular RNA circASS1 is downregulated in breast cancer cells MDA‐MB‐231 and suppressed invasion and migration. Epigenomics. 2019;11(2):199‐213. 30657346 10.2217/epi-2017-0167

[53] Yang R , Xing L , Zheng X , Sun Y , Wang X , Chen J . The circRNA circAGFG1 acts as a sponge of miR‐195‐5p to promote triple‐negative breast cancer progression through regulating CCNE1 expression. Mol Cancer. 2019;18(1):4. 30621700 10.1186/s12943-018-0933-7 PMC6325825

[54] Chen BO , Wei W , Huang X , et al. circEPSTI1 as a prognostic marker and mediator of triple‐negative breast cancer progression. Theranostics. 2018;8(14):4003‐4015. 30083277 10.7150/thno.24106 PMC6071524

[55] Liu H , Bi J , Dong W , et al. Invasion‐related circular RNA circFNDC3B inhibits bladder cancer progression through the miR‐1178‐3p/G3BP2/SRC/FAK axis. Mol Cancer. 2018;17(1):161. 30458784 10.1186/s12943-018-0908-8 PMC6245936

[56] Su H , Tao T , Yang Z , et al. Circular RNA cTFRC acts as the sponge of MicroRNA‐107 to promote bladder carcinoma progression. Mol Cancer. 2019;18(1):27. 30782157 10.1186/s12943-019-0951-0 PMC6379985

[57] Cao S , Chen G , Yan L , Li L , Huang X . Contribution of dysregulated circRNA_100876 to proliferation and metastasis of esophageal squamous cell carcinoma. Onco Targets Ther. 2018;11:7385‐7394. 30425526 10.2147/OTT.S177524 PMC6204868

[58] Rong J , Wang Q , Zhang Y , et al. Circ‐DLG1 promotes the proliferation of esophageal squamous cell carcinoma. Onco Targets Ther. 2018;11:6723‐6730. 30349305 10.2147/OTT.S175826 PMC6186307

[59] Yao T , Chen Q , Shao Z , Song Z , Fu L , Xiao B . Circular RNA 0068669 as a new biomarker for hepatocellular carcinoma metastasis. J Clin Lab Anal. 2018;32(8):e22572. 29785842 10.1002/jcla.22572 PMC6816935

[60] Shaker OG , Mohammed SR , Mohammed AM , Mahmoud Z . Impact of microRNA‐375 and its target gene SMAD‐7 polymorphism on susceptibility of colorectal cancer. J Clin Lab Anal. 2018;32(1):e22215. 10.1002/jcla.22215 PMC6817095 28374902

[61] Jemal A , Siegel R , Xu J , Ward E . Cancer statistics, 2010. CA Cancer J Clin. 2010;60(5):277‐300. 20610543 10.3322/caac.20073

[62] Greenhalgh J , Dwan K , Boland A , et al. First‐line treatment of advanced epidermal growth factor receptor (EGFR) mutation positive non‐squamous non‐small cell lung cancer. Cochrane Database Syst Rev. 2016;5:CD010383. 10.1002/14651858.CD010383.pub2 27223332

[63] Shi X , Ma C , Zhu Q , et al. Upregulation of long intergenic noncoding RNA 00673 promotes tumor proliferation via LSD1 interaction and repression of NCALD in non‐small‐cell lung cancer. Oncotarget. 2016;7(18):25558‐25575. 27027352 10.18632/oncotarget.8338 PMC5041926

[64] Zhu L , Ge J , Li T , Shen Y , Guo J . tRNA‐derived fragments and tRNA halves: the new players in cancers. Cancer Lett. 2019;452:31‐37. 30905816 10.1016/j.canlet.2019.03.012

[65] Tong C , Wu M , Cho W , To K . Recent advances in the treatment of breast cancer. Front Oncol. 2018;8:227. 29963498 10.3389/fonc.2018.00227 PMC6010518

[66] Antoni S , Ferlay J , Soerjomataram I , Znaor A , Jemal A , Bray F . Bladder Cancer Incidence and Mortality: a global overview and recent trends. Eur Urol. 2017;71(1):96‐108. 27370177 10.1016/j.eururo.2016.06.010

[67] Zhang N , Wen D , Shan B , et al. Clustering and geographic variation of upper gastrointestinal cancers in a high‐risk region of esophageal cancer in northern China. Asian Pac J Cancer Prev. 2011;12(1):193‐198. 21517256

[68] Wang Z . Not just a sponge: new functions of circular RNAs discovered. Sci China Life Sci. 2015;58(4):407‐408. 25680857 10.1007/s11427-015-4826-3

[69] Dou Y , Cha DJ , Franklin JL , et al. Circular RNAs are down‐regulated in KRAS mutant colon cancer cells and can be transferred to exosomes. Sci Rep. 2016;6:37982. 27892494 10.1038/srep37982 PMC5125100

[70] Greene J , Baird AM , Brady L , et al. Circular RNAs: biogenesis, function and role in human diseases. Front Mol Biosci. 2017;4:38. 28634583 10.3389/fmolb.2017.00038 PMC5459888

[71] Liu J , Liu T , Wang X , He A . Circles reshaping the RNA world: from waste to treasure. Mol Cancer. 2017;16(1):58. 28279183 10.1186/s12943-017-0630-y PMC5345220

[72] Yang Z , Xie L , Han L , et al. Circular RNAs: regulators of cancer‐related signaling pathways and potential diagnostic biomarkers for human cancers. Theranostics. 2017;7(12):3106‐3117. 28839467 10.7150/thno.19016 PMC5566109

[73] Wu J , Li G , Wang Z , et al. Circulating microRNA‐21 is a potential diagnostic biomarker in gastric cancer. Dis Markers. 2015;2015:435656. 26063956 10.1155/2015/435656 PMC4433679

[74] Li P , Chen H , Chen S , et al. Circular RNA 0000096 affects cell growth and migration in gastric cancer. Br J Cancer. 2017;116(5):626‐633. 28081541 10.1038/bjc.2016.451 PMC5344286

[75] Huang M , He YR , Liang LC , Huang Q , Zhu ZQ . Circular RNA hsa_circ_0000745 may serve as a diagnostic marker for gastric cancer. World J Gastroenterol. 2017;23(34):6330‐6338. 28974900 10.3748/wjg.v23.i34.6330 PMC5603500

[76] Li RC , Ke S , Meng FK , et al. CiRS‐7 promotes growth and metastasis of esophageal squamous cell carcinoma via regulation of miR‐7/HOXB13. Cell Death Dis. 2018;9(8):838. 30082829 10.1038/s41419-018-0852-y PMC6079012

